# Severity and long-term complications of surgical site infections after orthognathic surgery: a retrospective study

**DOI:** 10.1038/s41598-020-68968-2

**Published:** 2020-07-21

**Authors:** Anne-Sabine Cousin, Pierre Bouletreau, Joris Giai, Badr Ibrahim, Aurélien Louvrier, Nicolas Sigaux

**Affiliations:** 1 Department of Maxillofacial Surgery and Facial Plastic Surgery, Lyon Sud Hospital, Hospices Civils de Lyon, Claude Bernard Lyon 1 University, Pierre Bénite, France; 2 Department of Biostatisitics, Lyon Sud Hospital, Hospices Civils de Lyon, Pierre Bénite, France; 3 Department of Maxillofacial Surgery and Facial Plastic Surgery, Lyon Sud Hospital, Hospices Civils de Lyon, Pierre Bénite, France; 4 Department of Maxillofacial Surgery and Stomatology, Centre Hospitalier Régional Universitaire Jean-Minjoz, 25000 Besançon, France

**Keywords:** Infection, Oral manifestations

## Abstract

Surgical site infections (SSI) occur in 1.4% to 33.4% of cases after orthognathic surgery. This type of complication is a major concern to surgical teams, but there is no consensus for the prevention and treatment of SSI in orthognathic surgery. The purpose of this descriptive study was to evaluate the severity and the consequences of postoperative infections. The charts of all the patients operated on by the orthognathic surgery team between January 2015 and July 2017 were collected. All types of orthognathic procedures (Le Fort I maxillary osteotomy, bilateral sagittal split mandibular osteotomy, and genioplasty) were screened, and patients diagnosed with SSI were included. Demographic data, timing and severity of the infection, as well as long-term complications were recorded. Five hundred and twelve patients were screened. Forty-one patients (8%) presenting with SSI were included. There were 18 men and 23 women. The site of the infection was mandibular for 38 patients (92.7%) and maxillary for 3 patients (7.3%). The average time between surgery and infection was 31.5 days. Twenty-four patients received isolated oral antibiotics for inflammatory cellulitic reaction (58.8%), 15 patients had a localized collection treated by incision and drainage under local anesthesia (36.6%), and 2 patients had an extensive collection requiring surgical drainage under general anesthesia (4.9%). Five patients (12.2%) needed hardware removal for plate loosening, and 2 patients (4.9%) developed chronic osteomyelitis. Infection following orthognathic surgery is easily treated most of the time with no long-term complications. In cases of patients with potential risk factors for severe infection, antibiotics may be given with curative intents.

## Introduction

Orthognathic surgery is a common surgery with its own specific set of complications^1,2^. Surgical site infections (SSI) following orthognathic surgery occurs in 1.4 to 33.4% of cases. They are a major concern for surgical teams^3,4^. Preventive and therapeutic management is not standardized. The indication for antibiotic therapy or antibiotic prophylaxis in orthognathic surgery is still debated^5^. Practices vary widely amongst different teams with respect to the choice of antibiotic type and duration of treatment^6,7^. However commonly accepted good practice parameters mandate that antibiotic use should be kept to the minimum necessary to prevent bacterial resistance. Furthermore, there is no data in the literature regarding the severity and long-term complications of SSI in orthognathic surgery. Specifically, the rates of extensive cellulitis, osteomyelitis, pseudarthrosis and interruptive mandibulectomy remain unknown^8^. Our hypothesis was that single dose antibioprophylaxis was not associated with a high rate of severe postoperative complications. The purpose of this descriptive study was to measure the initial severity and the long-term consequences of postoperative infections following orthognathic surgery.

## Methods

We performed a retrospective screening of all the patients operated on for orthognathic surgery between January 2015 and June 2017 in our department of maxillofacial surgery. Our hypothesis was that the rate of severe postoperative infections was low. All patients with SSI were included. Orthognathic surgery was defined as any of the following procedures: bilateral sagittal split osteotomy (BSSO), Le Fort 1 osteotomy, bimaxillary osteotomy, intermaxillary disjunction, and genioplasty. Third molar extractions could be associated to any of these procedures. Infection was defined by any persistent inflammatory reaction after orthognathic surgery, such as swelling, pus or development of cellulitis. Episodes of infections following hardware removal were not included^5,9,10^. All patients were followed up for at least 6 months. Informed consent was obtained from all participants. This research was approved by the local ethics committee (Collège régional de Chirurgie Maxillo-Faciale) on May 2, 2019, and was performed in accordance with the guidelines reported in the ethical principles for medical research involving human subjects (World Medical Association Declaration of Helsinki).

### Surgical procedure

All patients were asked to stop smoking at least 10 days before surgery. They were operated on by senior surgeons, under general anesthesia and induced hypotension. The surgical site was prepared with povidone-iodine solution before surgery—including intra oral teeth and tongue brushing with the same solution. Standard peroperative IV prophylatic antibiotic were given to all patients, based on SFAR (Société Française d’Anesthésie Réanimation, *French Society of Anesthesia*) recommendations: intravenous AMOXICILLIN CLAVULANIC ACID 2 g was given during anesthesia induction, followed by 1 g every 2 h of operative time; in patients allergic to penicillin, intravenous CLINDAMYCIN 900 mg was given, followed by 600 mg every 4 h of operative time^11^.

Le Fort osteotomy was performed using a reciprocating saw or Piezotome. The maxilla was segmented if needed. Fixation of the maxilla and BSSO was obtained using titanium mini plates. Third molars were removed at the time of surgery if necessary. Surgical wounds were sutured with Ethicon coated VICRYL 3.0 sutures (Johnson and Johnson International).

Patients were kept in hospital for at least 2 nights after the surgery. A course of antibiotics was not routinely prescribed postoperatively. Radiographic control was performed on post-op day 1. All patients had postoperative intermaxillary elastic banding for 1 month. The patients were discharged on postoperative day 2 with a soft diet, analgesics, teethbrushing after each meal and 0.12% CHLOREXIDINE mouthwashes^12^. Standard post-operative care and oral hygiene instructions were given to each patients. They were seen in consultation at 2 weeks, 1 month, 3 months and 6 months postoperatively.

When SSI occured, antibiotics were prescribed for 10 to 15 days and oral hygiene was re-emphasized. The first line antibiotic was AMOXICILLIN CLAVULANIC ACID 1 g every 8 h. CLINDAMYCIN 600 mg every 8 h was the second line treatment in case of penicllin allergy. When the clinical and radiological evaluation demonstrated a collection, a surgical drainage was performed: local collection were drained under local anesthesia and irrigated with a normal saline solution. Extensive collections were drained under general anesthesia.

**Figure 1:**
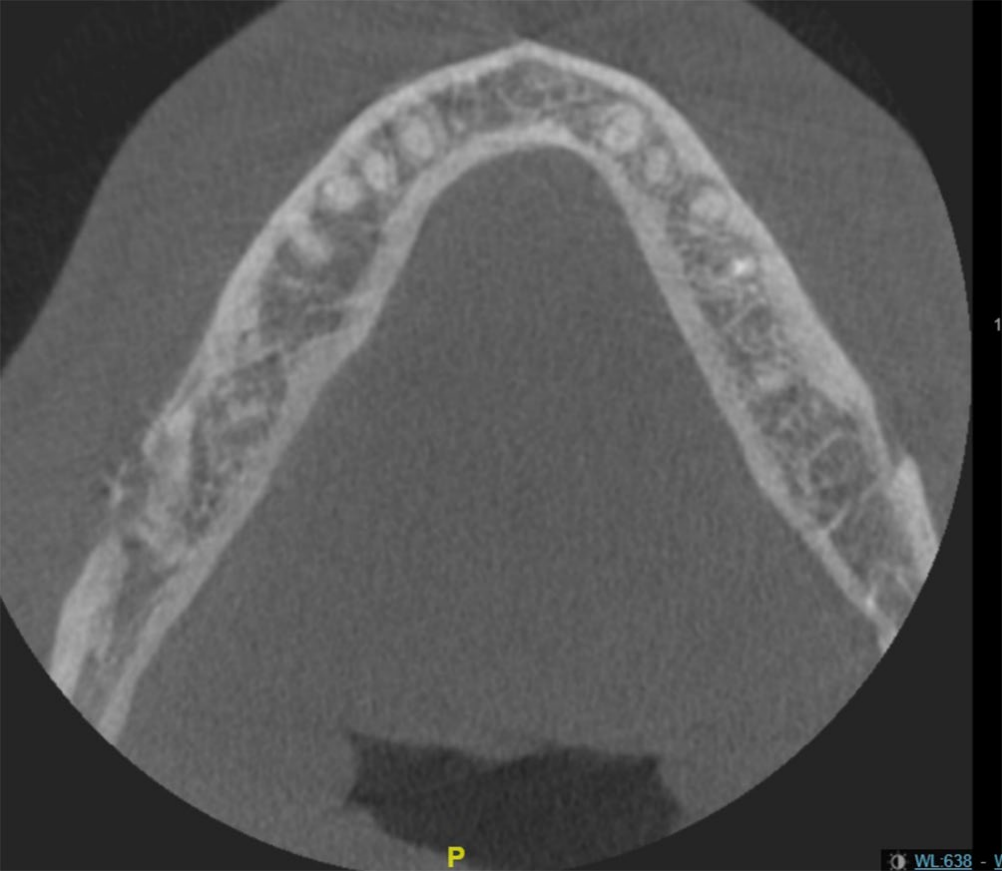
Axial view of Cone Beam CT: right mandibular osteomyelitis.

In case of osteomyelitis (Fig. 1), the patient was managed in collaboration with the infectious diseases department. Following surgical cleansing and bacteriological sampling, culture directed antibiotics were selected and administered for a period of 6 weeks to 3 months.

### Study variables

The following variables were collected: medical history (including tobacco use, diabetes, immunosuppressive treatment or pathology) age, sex, body mass index (BMI), type of surgery, time between surgery and infection, infection severity and local evolution.

### Data collection

Every complication occuring in the 6 months following surgery was documented. Antibiotic type, duration of treatment, surgical drainage and patient’s evolution were recorded.

The initial SSI severity was defined by the following presentations: cellulitic reaction treated with oral antibiotics, local collection requiring surgical drainage under local anaesthesia, and extensive cellulitis needing surgical drainage under general anesthesia.

The evolution of SSI was classified according to the following categories: complete recovery, plate loosening or pain requiring hardware removal, and long-term complications (osteomyelitis or pseudarthrosis). The diagnosis of osteomyelitis was based on clinical history, sampling of the bone and histopathological characteristics^13^.

### Statistical analysis

Categorical variables were described with frequencies and proportions, quantitative variables were described with median and interquartile range. The analysis was carried out using R Software version 3.5.0^14^.

## Results

### Patients demographic characteristics

**Figure 2:**
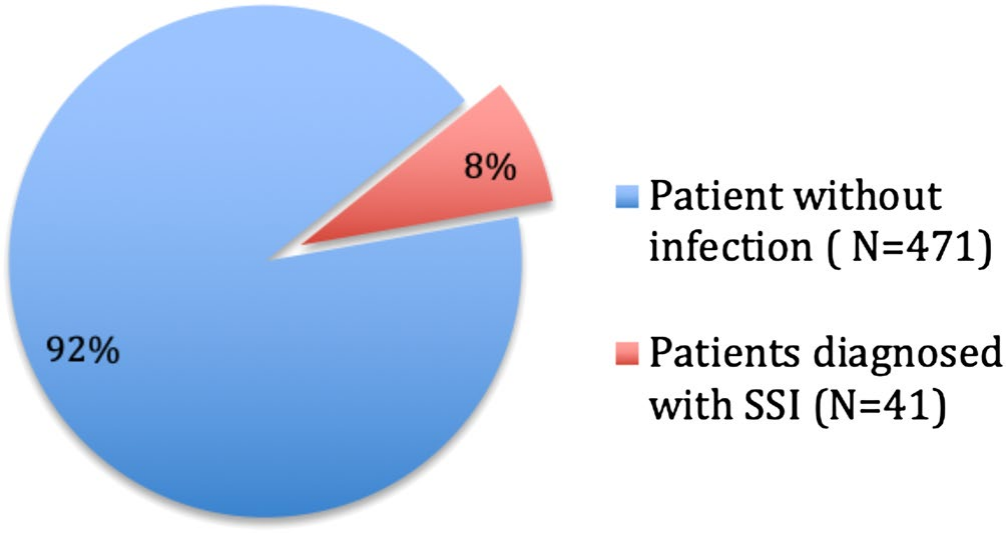
Schematic repartition of source population.

A total of 512 patients underwent orthognathic surgery between January 2015 and July 2017. The characteristics of the patients presenting with SSI are given in Table 1. A total of 41 patients (8%) were diagnosed with post-operative infection (Fig. 2). The age at the time of surgery ranged from 15 to 51 years old (median 20, mean 23.6). Nineteen patients had BSSO (46.3%), 21 had a bimaxillary surgery (51.2%), and one had isolated Le Fort 1 osteotomy (2.4%). The infection site was mandibular in 38 patients and maxillary in 3 patients. No infection was observed in genioplasty. Twenty-six patients were smokers (56%). The infection occurred on average 31.5 days and median 25 days after the surgery. No adverse effects of the antibiotics were reported.

**Table 1:** Demographic characteristics of patients presenting with SSI.

Outcome	Uncomplicated recovery	Hardware removal	Long-term complication	Total
	N = 34	N = 5	N = 2	N = 41
**Sex**				
F	20 (58.8%)	3 (60.0%)	1 (50.0%)	24 (58.5%)
H	14 (41.2%)	2 (40.0%)	1 (50.0%)	17 (41.5%)
**Age**				
Mean (SD)	23.41 (8.49)	19.60 (3.78)	37.50 (3.54)	23.6 (8.5)
Median (IQR)	19.5 (17–29)	18 (17–20)	37.5 (35–40)	20 (17–29)
Allergy to penicillin				
YES	3	0	0	3 (7.3%)
NO	31	5	2	38 (92.7%)
**Genioplasty **				
YES	5 (14.7%)	0 (0.0%)	0 (0.0%)	5 (12.2%)
NO	29 (85.3%)	5 (100.0%)	2 (100.0%)	36 (87.8%)
**Wisdom teeth**				
YES	8 (23.5%)	2 (40.0%)	0 (0.0%)	10 (24.4%)
NO	26 (76.5%)	3 (60.0%)	2 (100.0%)	31 (75.6%)
**Smoker**				
NO	23 (67.6%)	3 (60.0%)	0 (0.0%)	26 (63.4%)
YES	11 (32.4%)	2 (40.0%)	2 (100.0%)	15 (36.6%)
DIABETES				
NO	34	5	2	41 (100%)
YES	0	0	0	0
**Immunosuppression**				
NO	34	5	1	40 (97.6%)
YES	0	0	1	1 (2.4%)
**BMI (kg/m**^**2**^**)**				
Mean	20.6	22	24.9	21.04
N < 25	28 (82.3%)	4 (80%)	1 (50%)	33 (80.5%)
N = 25–30	6 (17.7%)	1 (20%)	1 (50%)	8 (19.5%)
N > 30	0	0	0	0
**Time interval from surgery to infection**				
Mean (SD)	31.32 (23.95)	26.40 (27.21)	47.50 (24.75)	31.5 (24.0)
Median (IQR)	25.5 (11–48)	24 (6–25)	47.5 (30–65)	25 (11–48)
Surgery				
Bimaxillary	19 (55.9%)	2 (40.0%)	0 (0.0%)	21 (51.2%)
BSSO	14 (41.2%)	3 (60.0%)	2 (100.0%)	19 (46.3%)
Le Fort 1	1 (2.9%)	0 (0.0%)	0 (0.0%)	1 (2.4%)
**Infection location**				
Mandible	31 (91.2%)	5 (100.0%)	2 (100.0%)	38 (92.7%)
Maxillary	3 (8.8%)	0 (0.0%)	0 (0.0%)	3 (7.3%)
**Initial severity of infection**				
Cellulitic reaction	20 (58.8%)	2 (40.0%)	2 (100.0%)	24 (58.5%)
Local collection	12 (35.3%)	3 (60.0%)	0 (0.0%)	15 (36.6%)
Extensive cellulitis	2 (5.9%)	0 (0.0%)	0(0.0%)	2 (4.9%)

### Infection severity

**Figure 3:**
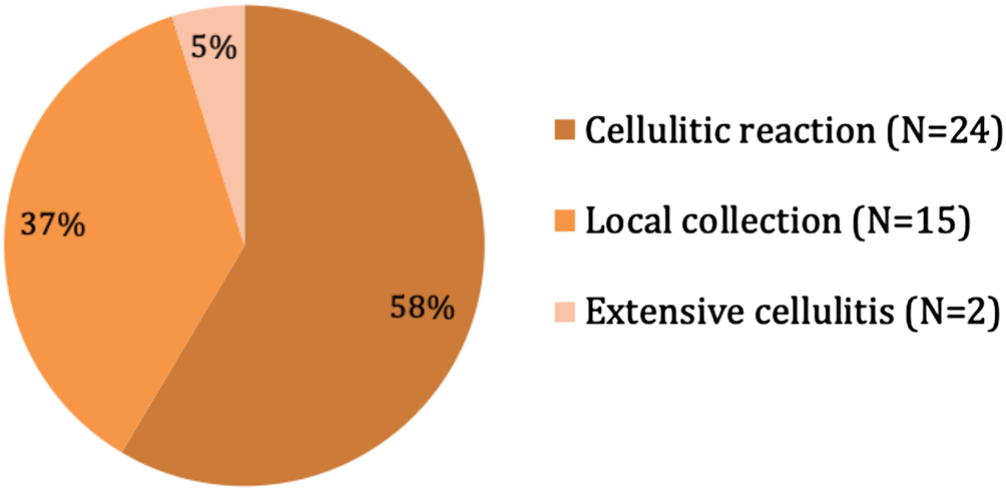
Initial severity of infections.

Twenty-four patients were treated only with antibiotics and mouthwashes for a cellulitic reaction (58.5%), 15 patients needed both oral antibiotics as well as an incision and drainage under local anesthesia (36.6%), and 2 patients required surgical drainage under general anesthesia for extensive cellulitis (4.9%)(Fig. 3). No patient required intensive care unit admission. Bacteriologic cultures and sensitivity testing on samples were only conducted for patients requiring drainage under general anesthesia.

### Local evolution

**Figure 4:**
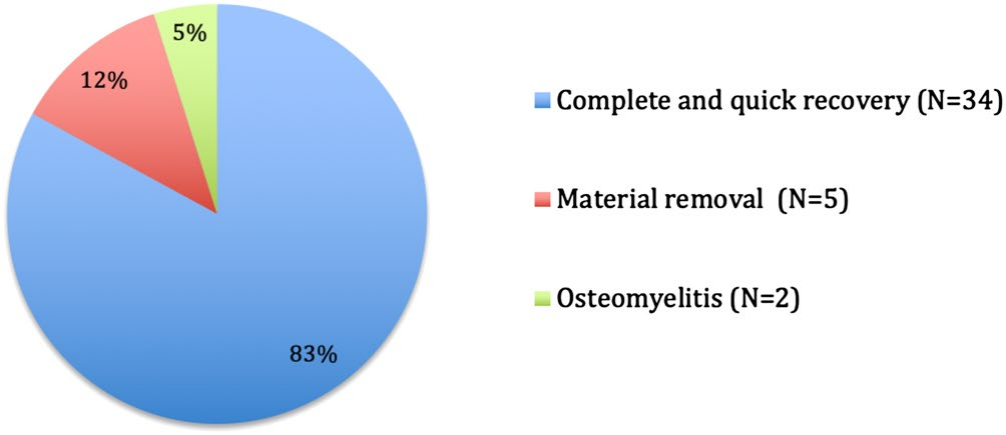
Local evolution after infection.

Thirty-four patients had a quick and complete recovery (82.9%). Five patients needed hardware removal for loose screws (12.2%). Two cases developed chronic osteomyelitis (4.9%) (Figs. 1 and 4). No case of pseudarthrosis was observed (Table 1).

**Figure 5:**
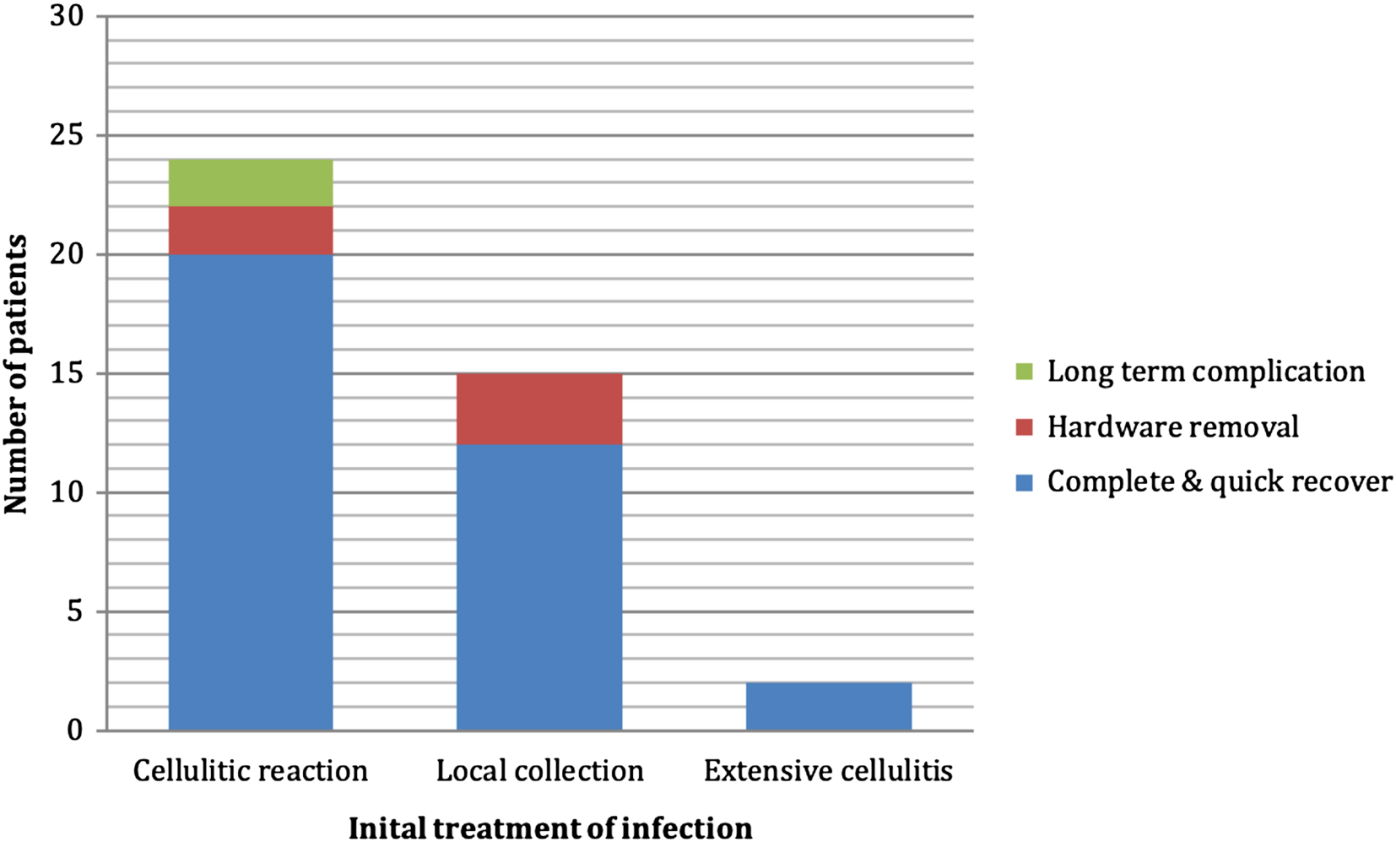
Local evolution according to initial presentation.

**Figure 6:**
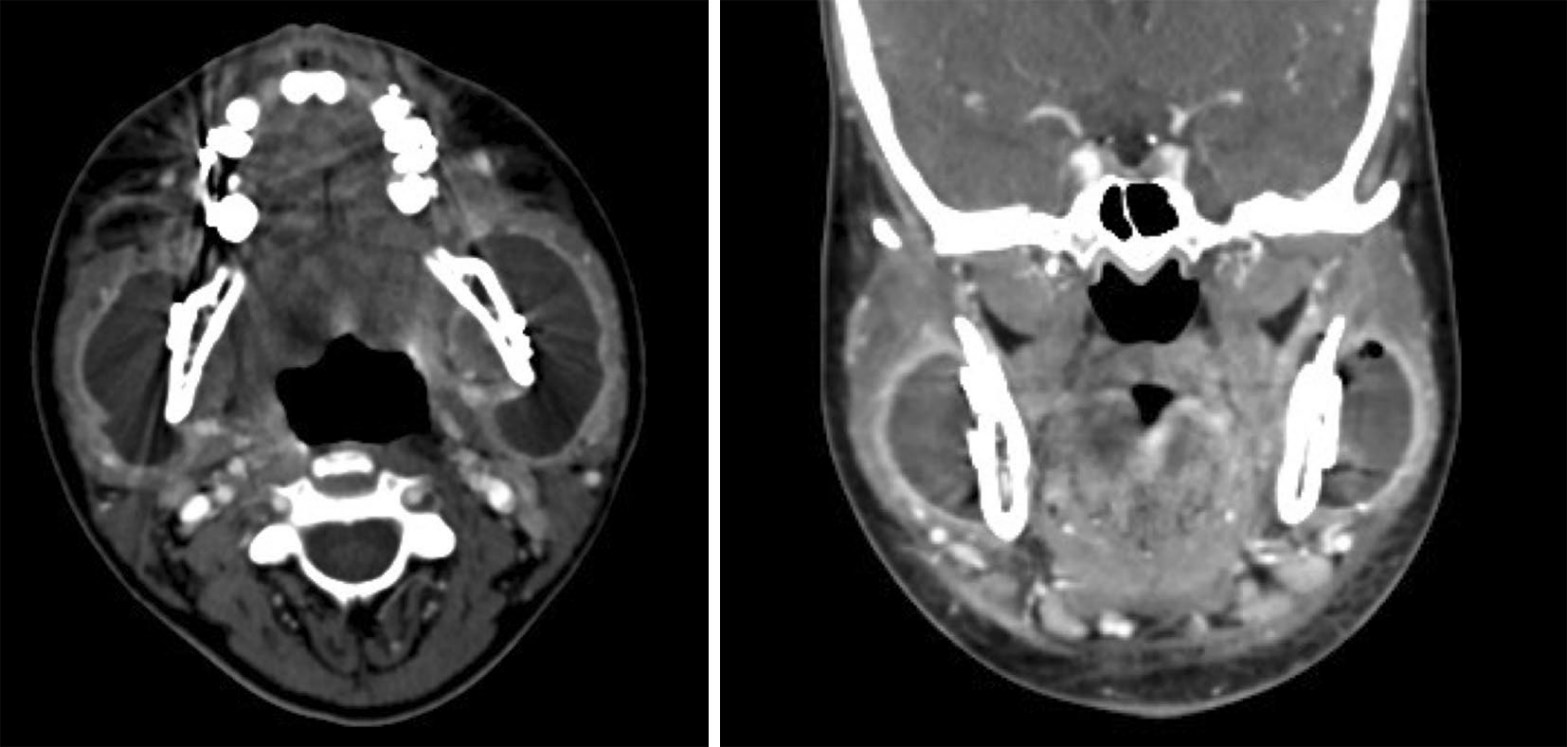
Extensive cellulitis with purulent collection after bilateral sagittal split osteotomy (CT scan, axial and coronal views).

The two patients who developed osteomyelitis were initially treated with a single antibiotic for a local cellulitic reaction. All patients who needed general anesthesia for extensive drainage had a complete and quick recovery (Figs. 5 and 6). Patients treated with CLINDAMYCIN all had a complete and quick recovery.

One of the two patients presenting with osteomyelitis was treated for rheumatoid arthritis with METHOTREXATE and ADALILUMAB. These treatments were discontinued 2 months before surgery (Fig. 1). The 2 cases of osteomyelitis resolved under medical treatment without requiring interruptive mandibulectomy.

## Discussion

According to this study, the overall rate of severe SSI is acceptable. These results are consistent with other published series in which a single prophylactic dose of AMOXICILLIN CLAVULANIC ACID was used^3,15,16^. Indeed, Chow et al. published the largest series in the literature on that topic and reported a rate of SSI of 7.4%, with a higher prevalence for mandibular location, compared with maxillary site^17–19^.

Orthognathic surgery is a clean contaminated surgery and is therefore expected to have a higher rate of SSI than non-contaminated surgery. As proven by Zijderveld et al., preoperative prophylaxis is known to decrease postoperative infections in orthognathic surgery^20^. Yet, there is significant heterogeneity regarding the choice of preoperative antibiotic in literature (Table 2). Barrier et al.noticed a higher rate of SSI when an antibiotic other than AMOXICILLIN CLAVULANIC ACID was used as prophylaxis^3^. There is evidence suggesting that AMOXICILLIN CLAVULANIC ACID is the best molecule for single dose pre-operative antibioprophylaxis^6,7,21–24^. These principles are in concordance with the French recommendations for antibiotic prophylaxis from the French Society of Anesthesia and Intensive Care Medicine (SFAR) which were respected in this study^11^.

**Table 2:** Reported strategies of antibiotics in the perioperative period.

Study	Study groups	Pre- and peri-operative antibiotic	Post-operative antibiotic	Infection rate	*p*
Lindeboom et al. (2003)^29^	1 dose regimen 4 doses regimen	Clindamycin 600 mg intravenously (IV), 15 min preoperatively Clindamycin 600 mg intravenously, 15 min preoperatively	Saline solution intravenously, every 6 h for 24 h Clindamycin 600 mg intravenously, every 6 h for 24 h	5.71% (2/35) 2.9% (1/35)	*p* > 0.05
Barrier et al. (2009)^3^	1 dose group	Amoxicillin 1 g intravenously 30 mn preoperatively and every 2 h perioperatively	none	7.0% (10/143)	
Chow et al. (2007)^17^		Penicillin and non penicillin antibiotics prophylaxis	none	7.4% (96/1,294)	
Ghantous et al. (2019)^28^	Intervention group	Amoxi-clav 1 g perioperatively	0.09% NaCl, 50 mL, every 8 h for 5 days Amoxi-clav 1 g, every 8 h for 5 days	2.5% (1/40) 0% (0/38)	*p* = 0.10
Tan et al. (2011)^23^	Oral group Intraveinous group	Ampicillin 1 g intravenously, and 500 mg every 6 h	Amoxicillin 500 mg every 8 h + NaCl every 6 h during 2 days. Then amoxicillin 500 mg every 8 h during 3 days Ampicillin 1 g every 6 h + oral lactose every 8 h during 2 days. Then amoxicillin 500 mg every 8 h during 3 days	14.1% (3/21) 28.6% (6/21)	*p* = 0.45
Zijderveld et al. (1999)^20^	Intervention group Comparison group	Amoxicillin clavulanate 2200 mg intravenously (30 mn preoperatively) OR cefuroxime 1,500 mg i.v. (30 min preoperatively) 0.9% sodium chloride i.v. (30 min preoperatively)		11–18% 53%	*p* < 0.004
Ruggles et al. (1984)^33^	Short term antibiotherapy Long-term antibiotherapy	Procaine penicillin 600.00 U and penicillin G 400,000 U intravenously 1 h preoperatively; penicillin G 2 g every 30 min perioperatively	Penicillin G 2 g 3 h Postoperatively Penicillin G 2 g every 6 h for 5 days	15% (3/20) 0% (0/20)	
Baqain et al. (2004)^34^	Short-term antibiotherapy Long-term antibiotherapy	Amoxicillin 1 mg intravenously at induction or Clindamycin 300 mg intravenously at induction	Amoxicillin 500 mg or clindamycin 150 mg 3 h postoperatively Amoxicillin 500 mg every 8 h or clindamycin 150 mg every 6 h for 5 days	23.5% (4/17) 11.7% (2/17)	*p* > 0.05

The treatment duration has been debated. Several authors showed that infection rates were lower with systematic extended postoperative antibiotics^4,16,22,25–27^. Nevertheless, these findings were recently challenged by Ghantous et al.in a prospective randomized double blind placebo controlled clinical trial^28^. Lindeboom et al.reported similar findings in a prospective randomized trial evaluating the SSI rates with CLINDAMYCIN (Table 2)^29,30^.

Excessive use of antibiotics carries its own sets of complications^31^. A postoperative antibiotic course is not recommended after surgery in international literature^6,11^. Short-term prophylaxis reduces adverse effects, decreases selection of resistant bacterial strains, and is more cost effective. Thus, systematic antibiotic treatment cannot be recommended given the available evidence.

Overall, the low rate of local complications as well as the plethora of possible adverse effects from extended antibiotic therapy support the indication for single dose preoperative antibioprophylaxis.

Of note, the delay between the surgery and the development of an infection could be surprising with an average of 31.51 days. This delay is however consistent with other published data. For instance, in the study of Chow et al., the delay between surgery and infection ranged from 3 to 4 weeks postoperatively^17^. The delay found in the current study might be overestimated. Indeed, the date of infection was noted as the date at which the patient presented out medical attention on our service.

It must be highlighted that the two patients presenting with osteomyelitis were smokers and showed a higher mean delay between surgery and infection (47.5 days vs 30 days; Table 1). The relationship between active tobacco use and the increased risk of infectious complications is well established in the orthopedic surgery literature^32^. Furthermore, the patients treated for long-term complications in our series were older with an average age of 37.5 years as opposed to 19.6 years for patients needing only hardware removal and 23.4 years for those recovering promptly following oral antibiotics only in the rest of the series. Moreover, one patient was immunosuppressed, which is a major risk factor of infection.

**Figure 7:**
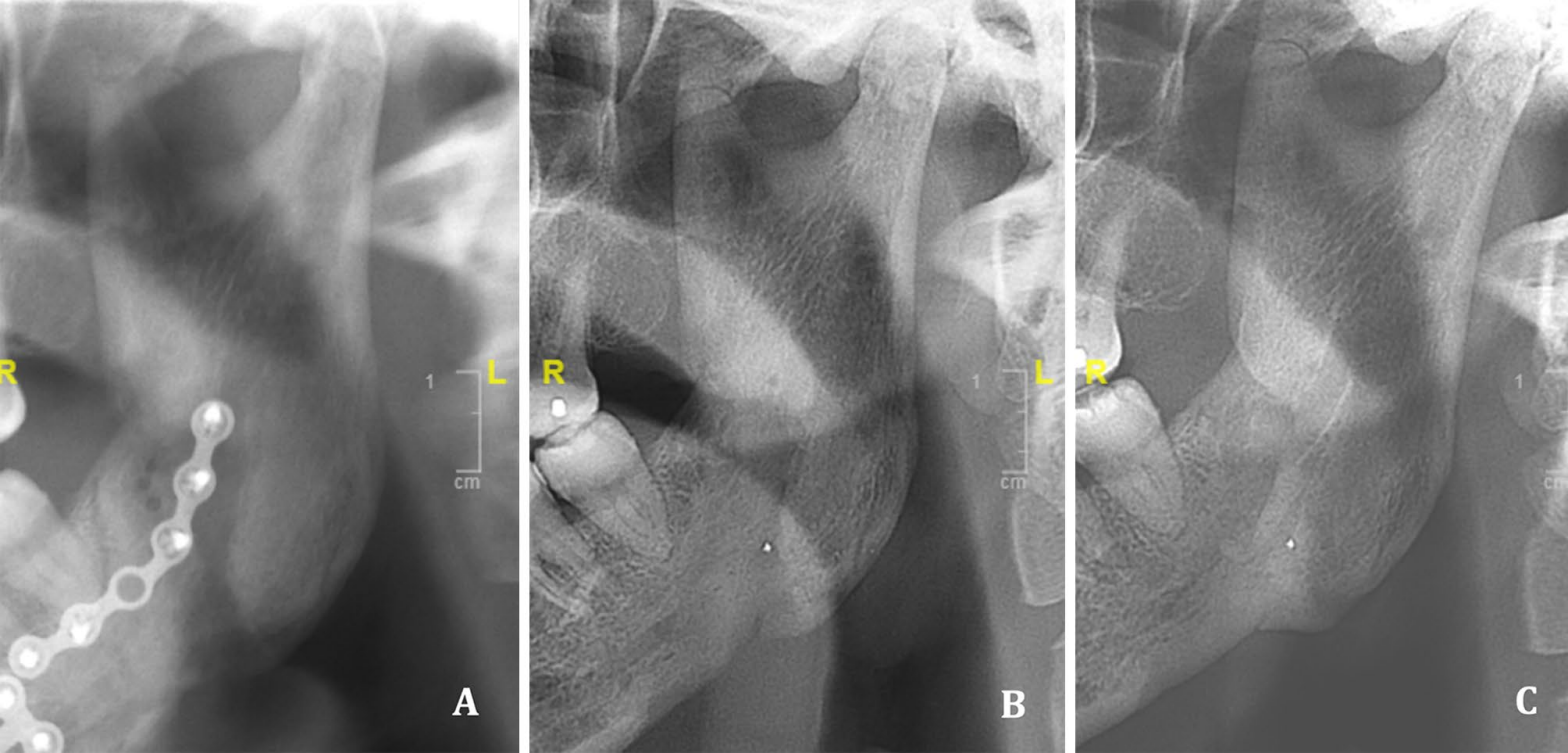
Radiographic evolution of osteomyelitis: diagnosis (**A**), 1 day after hardware removal and surgical curettage (**B**), and 1 year after the antibiotic treatment (**C**).

These results suggest that smokers, immunosuppressed patients and patients over 35 years old should be followed more closely in the postoperative period, looking for osteomyelitis (Fig. 7). A larger comparative series would be useful to specifically determine the risk factors of severe SSI. The indication for systematic extended postoperative antibiotic therapy for identified high-risk patients could be discussed.

## Data Availability

The dataset analyzed for this study is available upon request to the corresponding author.
